# Quantitating the Specificity and Selectivity of Gcn5-Mediated Acetylation of Histone H3

**DOI:** 10.1371/journal.pone.0054896

**Published:** 2013-02-21

**Authors:** Yin-Ming Kuo, Andrew J. Andrews

**Affiliations:** Department of Cancer Biology, Fox Chase Cancer Center, Philadelphia, Pennsylvania, United States of America; National Institute for Medical Research, Medical Research Council, United Kingdom

## Abstract

Lysine acetyltransferases (KATs) play a unique role in regulating gene transcription as well as maintaining the epigenetic state of the cell. KATs such as Gcn5 and p300/CBP can modify multiple residues on a single histone; however, order and specificity of acetylation can be altered by factors such as histone chaperones, subunit proteins or external stimulus. While the importance of acetylation is well documented, it has been difficult to quantitatively measure the specificity and selectivity of acetylation at different residues within a histone. In this paper, we demonstrate a label-free quantitative high throughput mass spectrometry-based assay capable of quantitatively monitoring all known acetylation sites of H3 simultaneously. Using this assay, we are able to analyze the steady-state enzyme kinetics of Gcn5, an evolutionarily conserved KAT. In doing so, we measured Gcn5-mediated acetylation at six residues (K14>K9 ≈ K23> K18> K27 ≈ K36) and the catalytic efficiency (k_cat_/K_m_) for K9, K14, K18, and K23 as well as the nonenzymatic acetylation rate. We observed selectivity differences of up to −4 kcal/mol between K14 and K18, the highest and lowest measurable k_cat_/K_m_. These data provide a first look at quantitating the specificity and selectivity of multiple lysines on a single substrate (H3) by Gcn5.

## Introduction

Many of the major human diseases, from cancer to heart disease, correlate with global changes in the identity and residue location of histone post-translational modifications (PTMs), supporting their central roles in maintaining the epigenetic state of the cell [1]–[4]. Changes in global acetylation patterns result from the diverse functions of histone or lysine acetyltransferases (KATs) [5], [6]. KATs play active roles in transcription, marking locations of DNA repair, and correlating to changes in the cell cycle [7], [8]. While the importance of the changes to the location and amount of acetylation and other modifications that make up the histone code is well supported [1], [9], [10], we have a limited understanding of how it is written.

Complicating our understanding of how the histone code is written is that many of the characterized KATs can modify histones at multiple lysines on a single histone. Both p300/CBP (KAT3A/B) and Gcn5 (KAT2A) acetylate multiple lysines on histone H3 [11]–[16]. The primary site of acetylation by Gcn5 is H3K14, but other specific sites are also modified, such as H3K9, H3K18, H3K23, and H3K27 [17]. Despite their critical importance, little is known about the specificity of Gcn5 for acetylation at lysine sites other than H3K14. Additionally, the field has been limited by an inability to quantitatively describe multiple modifications on a single protein.

Specificity is the ability of an enzyme to acetylate a discrete residue on H3, while selectivity is the efficiency of the enzyme to acetylate one position relative to another. In order to quantitate specificity and selectivity of specific lysines on a single protein, we need a method for monitoring acetylation on each lysine residue as a function of time. Site-specific antibody detection requires one antibody for each location and suffers from limitations such as epitope occlusion, which hinders the accuracy of quantitative measurements. This makes it almost impossible to have full coverage of multiple residual modifications via antibody assays. Alternately, radioactive or fluorescence labeling can only measure total acetylation. To overcome these limitations, we developed a label-free quantitative high throughput mass spectrometry method that quantitates the amount of all known sites of acetylation in a single run. Here we demonstrate the use of this assay to characterize the kinetic differences in specificity and selectivity between Gcn5-mediated and nonenzymatic lysine acetylation. Based on the observed catalytic efficiency, we have developed an enzyme specificity and selectivity model describing Gcn5 lysine acetylation.

The experimental and theoretical framework described in this paper not only allows for a more detailed understanding of Gcn5 but also provides a methodology for studying how other KATs acetylate multiple positions on histone H3 and other more complex histone complexes. By developing a robust and expandable assay for measuring histone acetylation we can provide the kinetic parameters not only on primary sites of acetylation but also on the secondary sites shown to be critical *in vivo* but not previously characterized by traditional assays. Our data suggest that Gcn5 is capable of efficiently acetylating positions K9, K18, and K23 in addition to H3K14. However, these positions are less catalytically efficient than K14, which makes them more susceptible to variations in acetyl-CoA levels, which can result from external factors such as a result of changes in metabolism [18].

## Materials and Methods

### Reagents

All Chemicals were purchased from Sigma-Aldrich (St. Louis, MO) or Fisher (Pittsburgh, PA), and the purity is the highest commercial grade or meets LC/MS grade. Ultrapure water was generated from a Millipore Direct-Q 5 ultrapure water system (Bedford, MA). Recombinant histones H3 and H3K14ac were purified and provided from the Protein Purification Core at Colorado State University (http://planetprotein.colostate.edu; for details see [19], [20]). Human recombinant Gcn5 enzyme was purchased from BPS Bioscience, lot # 110329 and 111031 (San Diego, CA). Acetyl-CoA was obtained from Sigma-Aldrich. Synthetic peptides (acetylated and propionylated) of high purity (>98%) were purchased from JPT peptide technologies (Acton, MA).

### Chemical quench

This assay demands an efficient quenching reagent to prevent the overestimation of acetylation rates. Although denaturants are capable of quenching the reaction, excess salts and acetyl-CoA have to be removed for MS analysis. Thus, an ideal reagent would be compatible with protein precipitation. Three different reagents (trichloroacetic acid (TCA) [21], isopropanol, and acetone [22]) were tested for the quenching efficiency. In order to determine the best quenching reagent, we mixed 180 nM Gcn5 and 200 µM acetyl-CoA (identical buffer conditions) with 0.8 µM H3 in 1/3 volumes of TCA (on ice), 2 volumes of isopropanol (on ice), or 4 volumes of acetone (−20°C) and incubated them overnight at 4°C. Both isopropanol and acetone resulted in an extra 2–9% acetylation. TCA was the only quench that resulted in no observable acetylation (data not shown).

### Enzymatic kinetics assays for Gcn5

Steady-state and single turnover kinetics for H3 and H3K14ac were performed under identical buffer conditions (100 mM HEPES buffer (pH 6.8) and 0.08% Triton X-100) at 37°C. Histone H3 concentrations were determined by the measurements of OD_276_ (ε_276_ = 4040). Steady-state (E<<S) assays contained 0.02 to 0.18 µM Gcn5, 0.15–45 µM H3 or H3K14ac, and 0.1–200 µM acetyl-CoA. Single turnover (E>>S) assays contained saturating 3 µM Gcn5, 0.5 µM H3 and 200 µM acetyl-CoA. At varying time points, assays were quenched/precipitated with 25% 4°C TCA, and the precipitate was then washed twice with 150 μL acetone (−20°C)[23]. Samples were dried, 1.5 μL propionic anhydride was added, and ammonium hydroxide was used to quickly adjust the pH to ∼8 [24]. Samples were then incubated at 51°C for 1 h followed by trypsin digestion (overnight at 37°C). In addition, nonenzymatic experiments [25]were conducted under the aforementioned assay procedures in the presence and absence of Gcn5, with 12 µM histone H3 and 100–300 µM acetyl-CoA.

### UPLC-MS/MS analysis

A Waters Acquity H-class UPLC (Milford, MA) coupled to a Thermo TSQ Quantum Access (Waltham, MA) triple quadrupole (QqQ) mass spectrometer was used to quantify acetylated H3 peptides. The digested H3 peptides were injected to an Acquity BEH C18 column (2.1×50 mm; particle size 1.7 μm) with 0.2% formic acid (FA) aqueous solution (solution A) and 0.2% FA in acetonitrile (solution B). Peptides were eluted over 11 min at 0.6 mL/min and 60°C, and the gradient was programmed from 95% solution A and 5% solution B and down to 80% solution A and 20% solution B in 11 min. The mass spectrometric conditions were: electrospray voltage: +4 kV; sheath gas pressure: 45 psi; auxiliary gas pressure: 20 psi; ion sweep gas pressure: 2 psi; collision gas pressure: 1.5 mTorr; andcapillary temperature: 380°C.Selected reaction monitoring (SRM) was used to monitor the elution of the acetylated and propionylated H3 peptides. The detail transitions are shown in Table 1.

**Table 1:** Detection parameters of tryptic peptides from Histone H3.

	Precursor ion (m/z)	Product ions (m/z)	Collision energy (eV)	Retention time (min)
TK_a_QTAR	373.711	475.262, 645.367	16	0.30
TK_p_QTAR	380.706	475.262, 659.387	16	0.41
K_a_STGGK_a_APR	493.275	570.335, 728.404, 815.437	20	0.44
K_a_STGGK_p_APR	500.270	584.355, 742.424, 829.456	20	0.65
K_p_STGGK_a_APR	500.272	570.335, 728.404, 815.437	20	0.69
K_p_STGGK_p_APR	507.264	584.355, 742.424, 829.456	21	1.14
K_a_QLATK_a_AAR	535.819	659.383, 772.467	22	2.99
K_a_QLATK_p_AAR	542.814	673.402, 786.486	22	3.99
K_p_QLATK_a_AAR	542.816	659.383, 772.467	22	4.11
K_a_QLATK_p_AAR	549.809	673.402, 786.486	22	4.95
K_a_SAPATGGVK_a_K_a_PHR	520.627	579.336, 905.531, 1231.690	26	4.00
K_p_SAPATGGVK_a_K_a_PHR	525.290	579.336, 905.531, 1231.690	26	4.60
K_a_SAPATGGVK_a_K_p_PHR	525.292	593.355, 919.550, 1245.709	26	4.60
K_a_SAPATGGVK_p_K_a_PHR	525.294	579.336, 919.550, 1245.709	26	4.60
K_p_SAPATGGVK_p_K_a_PHR	529.953	579.336, 919.550, 1245.709	27	5.10
K_p_SAPATGGVK_a_K_p_PHR	529.955	593.355, 919.550, 1245.709	27	5.18
K_a_SAPATGGVK_p_K_p_PHR	529.957	593.355, 933.570, 1259.729	27	5.00
K_p_SAPATGGVK_p_K_p_PHR	534.616	593.355, 933.570, 1259.729	27	5.60
YQK_a_STELLIR	646.864	744.461, 831.493, 1001.598	25	8.31
YQK_p_STELLIR	653.859	744.461, 831.493, 1015.588	26	9.23
K_a_LPFQR	415.748	450.245, 547.298	17	5.64
K_p_LPFQR	422.742	450.245, 547.298	18	6.35
EIAQDFK_a_TDLR	689.354	288.203, 936.478	27	8.56
EIAQDFK_p_TDLR	696.349	288.203, 950.497	27	9.31
VTIMPK_a_DIQLAR	476.274	600.382, 715.409, 885.515, 982.567	24	11.13
VTIMPK_p_DIQLAR	480.938	600.382, 715.409, 899.534, 996.587	24	11.69

### QqQ MS data analysis

Each acetylated and/or propionylated peak was identified by retention time and specific transitions (Table 1). The resulting peak integration was done using Xcalibur software (version 2.1, Thermo). The fraction of a specific peptide (F_p_) is calculated by eq. 1, where I_s_ is the intensity of a specific peptide state and I_p_ is the intensity of any state of that peptide [26], [27]. For
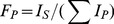
(1)


example, the fraction of K_a_STGGK_a_APR (K9 to R17) can be calculated by the intensity (integrated area) of K_a_STGGK_a_APR divided by the summed intensities of K_a_STGGK_a_APR, K_p_STGGK_a_APR, K_a_STGGK_p_APR, and K_p_STGGK_p_APR which are all possible states of this peptide (subscript a and p are acetylation and propionylation, respectively). The fraction for a specific residue acetylated can be calculated as the sum of all F_p_ that contain that acetylated residue. For example the acetylation of K14 can be obtained by summation of the fractions of K_a_STGGK_a_APR and K_p_STGGK_a_APR. In order to calculate the fraction of acetylation for two or more positions (*e.g.* total fraction acetylation), the individual acetylation fractions are added up and divided by the number of lysines. To determine the total amount of acetylation, the sum of the fractions were multiplied by the initial concentration of H3.

### Data analysis

**Figure 1:**
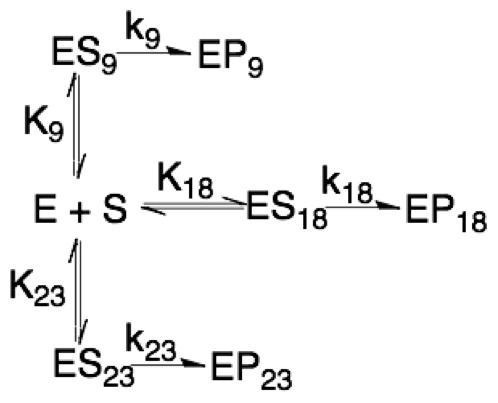
Scheme 1, three competing sites on one substrate (H3).



All models were fit to the data with Prism version 5.0d. The initial rates (*v*) of acetylation were calculated from the linear increase in acetylation as a function of time prior to a total of 10% of the sum of all residues being acetylated. To measure steady-state parameters for acetyl-CoA, the initial rates were calculated based on time where less than 10% of the acetyl-CoA was consumed (based on a coupled assay [28])and where the acetylated H3 fraction mediated by Gcn5 is less than 0.1 times the fraction of unacetylated H3. For K14 acetylation, the steady-state parameters k_cat(app)_, K_(app)_(*i.e.* K_m(app)_ or K_1/2_), and Hill coefficient (nH) were determined by fitting eq. 2, where [S] is the concentration of substrate (H3 or acetyl-CoA), and [E] is the concentration of Gcn5. The Hill coefficient (nH) was only



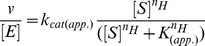
(2)


used when needed (titrating H3K14ac). Modeling acetylation beyond that of H3K14ac was done from scheme 1 (Fig. 1). Our data from equation two suggest that while the titration of acetyl-CoA will not require a Hill coefficient, the titration of H3K14ac has a Hill coefficient of approximately the same value for all three constant. Derivations of scheme 1 (Fig. 1) result in eq. 3, where *vx* is equal to initial velocity of the location of interest, A is equal to the multiplication of the three Ks divided by the K of interest (eq. 4),

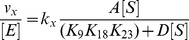
(3)




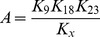
(4)

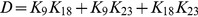
(5) and D is equal to eq. 5. In this mechanism (k_cat_/K_1/2_^nH^)_(app)_ from eq. 2 becomes eq. 6, where k_x_ is the rate of product formation for the residue of interest and K_x_ is equal to the K of interest.



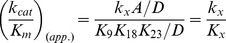
(6)The catalytic proficiency for specific lysine residue was calculated using eq. 7, where R is the gas.



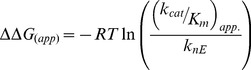
(7)constant, T is the absolute temperature, (k_cat_/K)_(app)_ is catalytic efficiency for the residue of interest, and k_nE_ is the second order rate constant for nonenzymatic acetylation. When acetyl-CoA is in an excess amount, nonenzymatic acetylation was described by a single exponential eq. 8,



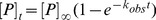
(8) 8where [P]_t_ is the concentration of specific acetylated lysine at time (t), which was determined by multiplying the known concentration of initial substrate and the fraction of specifically acetylated lysine, t is time, and k_obs_ is the observed rate. The second order rate constant for nonenzymatic acetylation was determined by eq. 9, where k_obs_ is the pseudo-first order rate of acetylation from eq. 8, [acCoA] is the concentration of acetyl-CoA, k_nE_ is the second order rate constant, and b is the y-intercept.



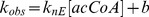
(9)


## Results

### Validation of quantitative analysis of H3 acetylation (label-free quantitative assay)

Before we can begin to understand the kinetics of acetylating multiple positions on a single histone, we need to validate our assay. This assay relies on three basic steps: a quench to stop the reaction (See experimental procedures), digestion to obtain fragments optimal for MS, and UPLC-MS/MS. All three of these steps need to be optimized, but quantitative MS analysis is critical for optimization.

#### Histone digestion

**Figure 2:**
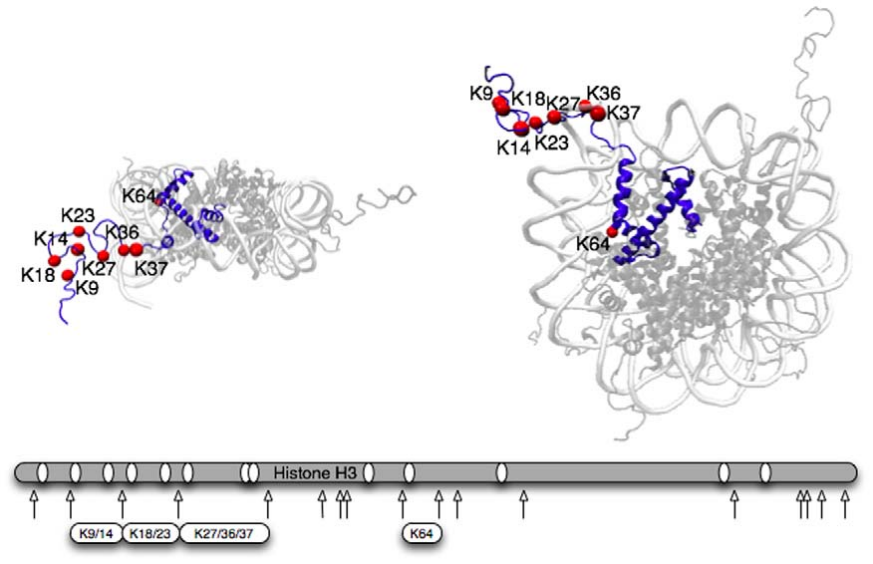
Structure of H3 (blue highlight) in nucleosome, which is constructed from the PDB 1KX5 nucleosome structure [54]. The red dots show the lysine locations that are reportedly acetylated in this study. The grey bar represents one H3 sequence and the white ovals show the relative lysine sites and arrows are the location of arginines that are the only locations digested by trypsin after chemical propionylation or acetylation.

There are two considerations for the tryptic digestion of histones: first, the large numbers of lysines and arginines can result in peptides too small to accurately detect. Second, once lysines are acetylated, they are no longer amiable for digestion. This leaves two options for digestion; chemically alter the lysines or use Arg-C, both of which results in only digesting at arginine residues. Both of these methods have been used with success for studying histone modifications [29], [30]. However, Arg-C requires the use of both calcium and surfactants to approach the efficiency of trypsin. Therefore, we chose to propionylate the histone prior to the addition of trypsin. This protocol has been successfully applied to identify and quantify histone PTMs for different research subjects [31]–[33]. Both chemical propionylation and/or acetylation as a result of the assay will prevent cleavage by trypsin, resulting in the same proteolytic peptides as the Arg-C digestion (Fig. 2). Thus, either unacetylated or acetylated lysines will remain on individual peptides. This avoids loss of detection for very short peptides generated by trypsin alone and neutralizes the charge and increases the hydrophobicity at the unacetylated lysine residuals, providing greater separation on the C-18 column. Thus propionylation reduces the number of experimental steps and contributes to higher reproducibility and simplification of the data processing.

#### Selected reaction monitoring (SRM) and quantitation

QqQ MS provides unmatched sensitivity, dynamic range, and quantitative capability. SRM allows us to measure specific parent ion to product ion transitions that are both unique to the peptide of interest and to the site of modification (Table 1). We used a series of synthetic peptides in direct infusion experiments for the optimization of collision parameters and to determine which transitions provide the highest intensity for the acetylated and propionylated lysines of interest. The use of synthetic peptides also demonstrates that different acetylation or propionylation patterns within one peptide fragment have the same ionization efficiency. Mixing different ratios of acetylated to propionylated (non-acetylated) peptides followed by QqQ MS, we were able to plot the measured fraction of observed acetylation verses the actual acetylation ratio and observed a slope of 0.99 and an R2 = 0.998 (data not shown). This validates both our assumption and others who have used this approach to quantitate histone modifications [31]–[33]. This allows us to determine the fraction of modification and to confirm that we can indeed measure the low levels of acetylation needed for enzyme kinetics.

### Residue specific activity of Gcn5

The majority of residues acetylated by Gcn5 are located on the tail of histone H3 (*e.g.* K9, K14, K18, and K23) (Fig. 2), and even small peptide substrates commonly used in kinetic assay have multiple possible locations of acetylation. Therefore, an ideal assay should quantitate all known locations of acetylation. Herein we present a workflow that solves these problems and allows for the study of residue-specific enzyme kinetics.

**Figure 3:**
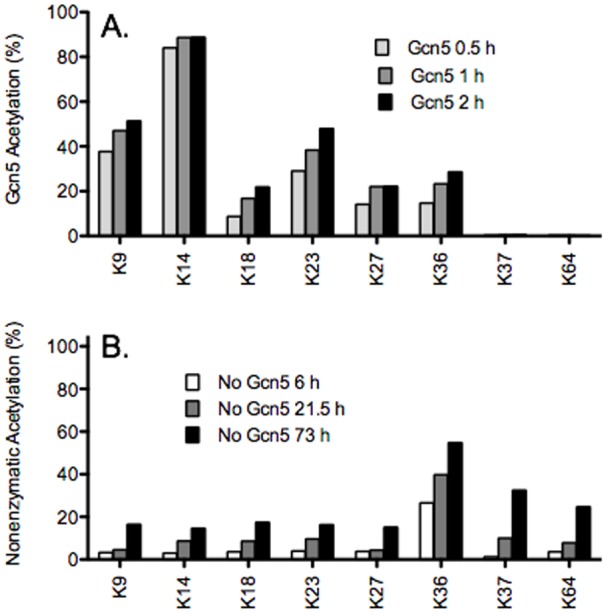
Sites acetylated on histone H3. (A) Gcn5 mediated acetylation of H3 (12 µM H3, 200 µMacetyl-CoA) at 0.5 (light grey), 1 (dark grey), and 2 (black) hours. (B) Nonenzymatic acetylation of H3 (12 µM) by acetyl-CoA (200 µM) at 6 (white), 21.5 (grey), and 73 (black) hours.

In order to determine the likely preference of acetylation, we first measured acetylation at several time points up to 2 hours (Fig. 3A). These data confirmed earlier studies [17], [34] that K14, K9, and K23 were the primary acetylation sites at 1 hour. Overnight we also observed acetylation of K18, K27, and K36, but no observable acetylation of K4, K37, K56, K64, K79, and K122 was found. From these data we focused our kinetic studies to K9, K14, K18, K23, K27, and K36, although all SRMs for other lysines were collected.

### Nonenzymatic acetylation

In order to determine Gcn5-mediated acetylation, we also characterized the specificity of nonenzymatic acetylation. Note that in the nonenzymatic assay, there is no significant lysine acetylation detected at 0.5 h. However, at long time points (>6 hours) in the presence of high levels of acetyl-CoA (200 µM), we observed multiple locations of acetylation (Fig. 3B). Interestingly, the sites of acetylation were not random but had a degree of specificity that differed from that of Gcn5. The primary site of nonenzymatic acetylation is K36, followed by K37, both of which are closer to the core of H3. K64 is the third most nonenzymatically acetylated residue; is in the core of H3 and is not significantly acetylated by Gcn5. We observed no significant acetylation at locations K4, K79, and K122. The lack of acetylation on K4 is also a notable observation due to its location at the end of the histone tail. Together these data suggest that at least some level of structure or reduced conformational dynamics enhances nonenzymatic acetylation.

**Figure 4:**
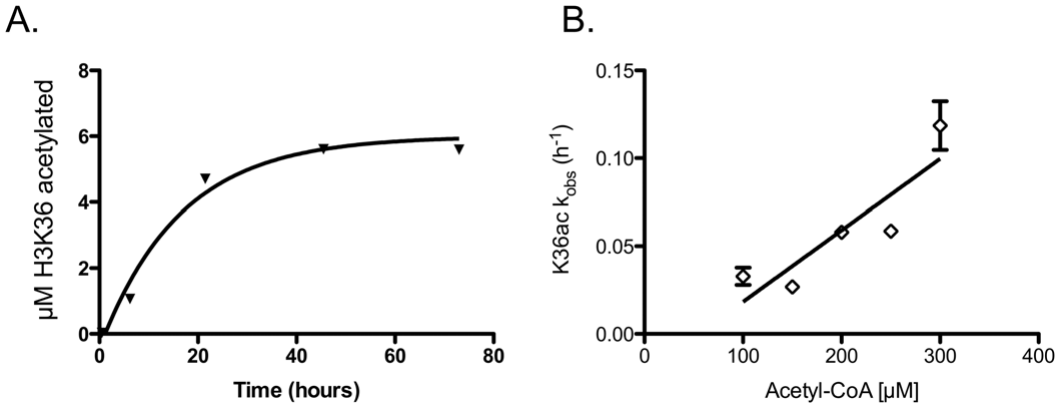
Second order rate constant for nonenzymatic acetylation. (A) Concentrations of acetylated H3K36 as a function of time fit to a pseudo-first order reaction ([acetyl-CoA] = 200 µM) with an apparent rate of 5.8±0.3×10^−2^ h^−1^. (B) k_obs_ for the nonenzymatic acetylation of K36 as a function of acetyl-CoA concentration resulting in an apparent rate constant of 4.1÷0.6×10^−4^ µM^−1^ h^−1^.

We next measured rates of nonenzymatic acetylation as a function of time at various concentrations of acetyl-CoA (Fig. 4). K36 is the first position to be acetylated and the only single position that acetylates to a definable endpoint of 58±5% of the total amount of histone (Fig. 4A). It is possible that the acetylation of K37 sterically inhibits the acetylation of K36 or that acetylation of the histone somehow alters the histone conformation, limiting acetylation. The acetylation rate of K36 defines the upper limit of how fast any single residue can be nonenzymatically acetylated (4.1 ± 0.6 x 10^-4^ µM^-1^ h^-1^) (Fig. 4B). Using these data (and eq. 8 and 9) we can calculate the expected amount of nonenzymatic acetylation for a given point in time and a set of reaction conditions. This calculation for K36 at a time of 1 hour under saturating conditions yields only 3–6%, where Gcn5 acetylates 23±7% of K36 in this time.

#### Monitoring histone acetylation by peptide and specific residue

**Figure 5:**
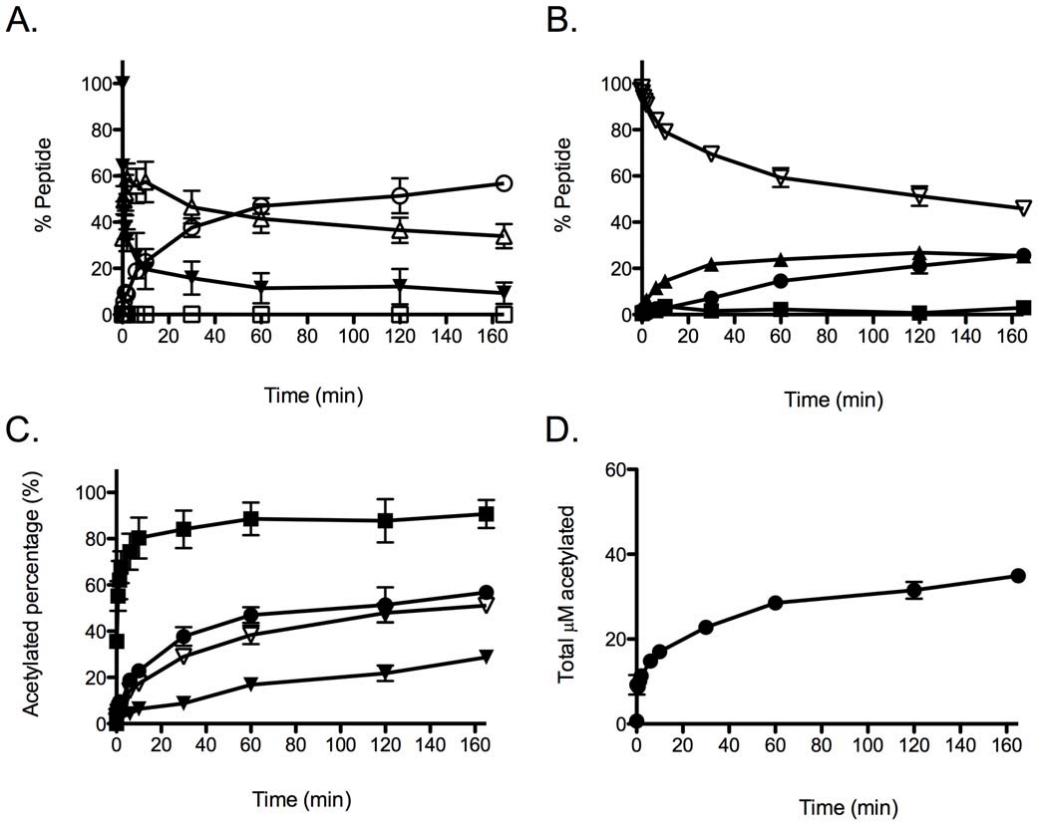
Multiple views of Gcn5-mediated H3 acetylation kinetics from bottom-up MS analysis, when [H3]  = 12 μM, [Gcn5]  = 180 nM, and [acetyl-CoA]  = 200 μM. (A) Changes of modifications on KSTGGKAPR: K_a_STGGK_a_APR (open circle), K_a_STGGK_p_APR (open square), K_p_STGGK_a_APR (open triangle), and K_p_STGGK_p_APR (solid reverse triangle). (B) Changes of modifications on KQLATKAAR: K_a_QLATK_a_AAR (solid circle), K_a_QLATK_p_AAR (solid square), K_p_QLATK_a_AAR (solid triangle), and K_p_QLATK_p_AAR (open reverse triangle). The data of (A) and (B) were directly obtained from MS SRM analysis. (C) Kinetics of fractions of acetylated K9 (solid circle), K14 (solid square), K18 (solid triangle), and K23 (open reverse triangle). (D) Kinetic of total acetylated lysine concentration on H3. The plots of (C) was generated from the calculation of (A) and (B). Apparently, K14 is the primary acetylation lysine by Gcn5 catalysis. While only the total or multiple acetylation is monitored, the acetylation amount from minor acetylation sites could be neglected, especially at short time points.

Monitoring histone acetylation by SRM provides a unique look at the kinetics of acetylation. There are three ways to visualize these data: we can plot the percentage of acetylation on each peptide, the total amount of acetylation at a specific lysine, and the total percent of acetylation. Monitoring the specific peptides, we can observe the appearance of H3K14 acetylation (KpSTGGKaAPR (K9 to R17)) followed by K9 and K14 both being acetylated (KaSTGGKaAPR). Because we never detected H3K9 acetylation by itself (KaSTGGKpAPR), the combined results demonstrate that H3K14 is acetylated prior to H3K9 (Fig. 5A). When we expand our analysis to include the other peptides, we also see acetylation of K23 occurring before K18 (Fig. 5B), suggesting the K14 is acetylated first, followed by K9 and K23, then K18. The rates of K27 and K36 acetylation are slower than K14, K9, and K23, making the determination of the direct order of acetylation difficult. By taking the sum of the fraction of KpSTGGKaAPR (K9 to R17) and KaSTGGKaAPR, we can monitor the total acetylation of K14 (Fig. 5C). To compute the amount of acetylation that would be observed by standard methods, we took the sum of the fraction of every acetylated peptide and multiplied it by the concentration of H3 (Fig. 5D). These data demonstrate how observed rates of total acetylation can be averaged out among those of individual residue acetylation. It is also likely that total acetylation under these conditions could be misinterpreted as biphasic or burst kinetics. This fact highlights the importance of this type of approach when dealing with full-length histones or substrate with multiple lysines.

#### Kinetics of initial H3 acetylation

**Figure 6:**
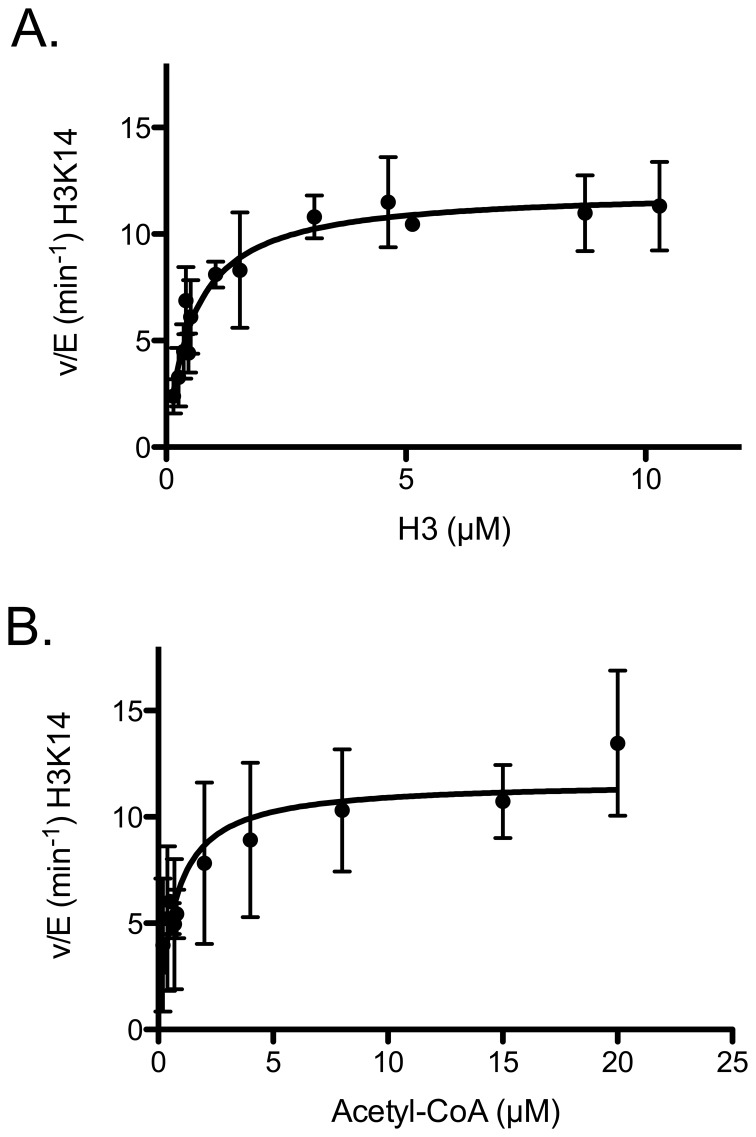
Determination of steady-state kinetic parameters for K14 acetylation by Gcn5. (A) k_cat_  = 12.1±0.1 min^−1^ and K_m_  = 0.5±0.02 µM are determined when [Gcn5]  = 18 nM, [acetyl-CoA]  = 200 μM with titrating 13 different H3 concentrations. (B) k_cat_  = 11.6±0.2 min^−1^ and K_m_  = 0.7±0.05 µM are determined when [Gcn5]  = 18 nM, [H3]  = 10 μM with titrating different acetyl-CoA concentrations.

To ensure that the experiments were under steady-state conditions, we limited our analysis to time points where the sum of the total fraction of acetylated histone was less than 10%. Under these conditions we did not observe any nonenzymatic acetylation, consistent with the estimate derived from the measured second order rate constant. We carried out a series of time course experiments under steady-state conditions, obtained the rate of initial 10% histone acetylation for different substrate concentrations, and then plotted v/E vs. substrate concentrations (Fig. 6). Using H3 as a substrate under these conditions, only K14 was sufficiently acetylated to determine the initial rate before >10% of H3 had at least one acetylation site. Other residues are acetylated prior to 10% total acetylation but could not be adequately quantitated with this substrate. The H3 steady-state parameters for K14 acetylation are Km(app) = 0.5±0.02 μM and kcat(app) =  12.1±0.1 min-1 (Fig. 6A and Table 2 and 3).

**Table 2:** Steady-state parameters of acetyl-CoA for Gcn5-mediated acetylation (mean ± standard error) of H3 (wt) and H3K14ac.

Substrate	Residue Acetylated	K_m(app)_ (K_1/2_)(µM)	*n*_H_	k_cat(app)_ (min^−1^)	(k_cat_/K_m_)_(app)_ (µM^−1^ min^−1^)	(k_cat_/K_1/2_ *^n^* ^H^)_(app) _(µM^−^ *n* ^H^min^−1^)	(k_cat_/K_m_)_(app)_/k_nE _(No Units)	(k_cat_/K_m_^nH^)_(app)_/k_nE _(µM^−nH+1^)
H3 (wt)	K14	0.5±0.02	*n.a.*	12.1±0.1	24.2±1.0	*n.a.*	(3.54±±0.48)×10^6^	*n.a.*
H3K14ac	K9	5.3±0.2	2.1±0.6	2.3±0.07	0.43±0.02	0.06±0.02	(6.35±0.89)×10^4^	(8.58±1.3)×10^3^
H3K14ac	K18	7.4±0.4	2.2±0.7	0.8±0.03	0.11±0.01	0.01±0.003	(1.58±0.23)×10^4^	(1.43±0.25)×10^3^
H3K14ac	K23	5.7±0.4	2.2±0.9	2.8±0.1	0.49±0.04	0.07±0.03	(7.19±1.08)×10^4^	(1.06±0.20)×10^4^
H3K14ac	K9&K18&K23	6.5±0.3	2.3±0.6	5.7±0.1	0.88±0.04	0.08±0.02	(1.28±0.18)×10^5^	(1.13±0.18)×10^4^

ΔΔG_(app)_ was calculated based on the (k_cat_/K_m_)_(app)_/k_nE_ where k_nE_ is the second order rate constant in µM^−1^ min^−1^ (6.83±0.89x10^−6^) for nonenzymatic acetylation (K36).

**Table 3:** Steady-state parameters of H3 (wt) and H3K14ac for Gcn5-mediated acetylation (mean ± standard error).

Substrate	Residue Acetylated	K_m(app)_ (K_1/2_)(µM)	*n*_H_	k_cat(app)_ (min^−1^)	(k_cat_/K_m_)_(app)_ (µM^−1^ min^−1^)	(k_cat_/K_1/2_ *^n^* ^H^)_(app) _(µM^−^ *n* ^H^min^−1^)	(k_cat_/K_m_)_(app)_/k_nE _(No Units)	(k_cat_/K_m_^nH^)_(app)_/k_nE _(µM^−nH+1^)
H3 (wt)	K14	0.5±0.02	*n.a.*	12.1±0.1	24.2±1.0	*n.a.*	(3.54±±0.48)×10^6^	*n.a.*
H3K14ac	K9	5.3±0.2	2.1±0.6	2.3±0.07	0.43±0.02	0.06±0.02	(6.35±0.89)×10^4^	(8.58±1.3)×10^3^
H3K14ac	K18	7.4±0.4	2.2±0.7	0.8±0.03	0.11±0.01	0.01±0.003	(1.58±0.23)×10^4^	(1.43±0.25)×10^3^
H3K14ac	K23	5.7±0.4	2.2±0.9	2.8±0.1	0.49±0.04	0.07±0.03	(7.19±1.08)×10^4^	(1.06±0.20)×10^4^
H3K14ac	K9&K18&K23	6.5±0.3	2.3±0.6	5.7±0.1	0.88±0.04	0.08±0.02	(1.28±0.18)×10^5^	(1.13±0.18)×10^4^

In cases where the Hill coefficient (*n*_H_) is greater than one, (k_cat_/K_1/2_ *^n^* ^H^)_(app)_ was also calculated. The units for (k_cat_/K_m_^nH^)_(app)_ are µM^−*n*H^ min^−1^ dividing by k_nE_ to determine (k_cat_/K_1/2_ *^n^* ^H^)_(app)_/k_nE_ (k_nE_  = 6.83±0.89×10^−6^ µM^−1^ min^−1^) results in µM to the power of the -*n*_H_+1 (µM^−nh+1−^). For this reason we can not calculate a ΔΔG_(app.)_ for anything other than K14ac (*n*_H_ = 1, ΔΔG_(app.)_  = −9.3±0.08 kcal mol^−1^).

In order to monitor the initial rate of acetylation as a function of acetyl-CoA concentration, we had to ensure the assays were under steady-state conditions. This is due to the fact that while we are monitoring the amount of H3 acetylated we are varying the amount of acetyl-CoA. In other words, when [H3]>[acetyl-CoA] we can only monitor the amount of acetylated H3 over time up to a concentration <10% [acetyl-CoA], but when [H3]<[acetyl-CoA] we can measure up to <10% [H3]. Under these conditions, the acetyl-CoA steady-state parameters for K14 acetylation are K_m(app)_ =  0.7±0.05 μM and k_cat(app)_ = 11.6±0.2 min^−1^ (Fig. 6B and Table 2 and 3).

To confirm that K14 is the first site acetylated on H3, we measured the single turnover kinetics under limiting H3 conditions and excess Gcn5 and acetyl-CoA. Under these conditions, H3 exist as H3-Gcn5 and >98% of K14 is acetylated prior to K9 or K23 (data not shown). Sequentially, K9 and K23 were the next acetylation sites, which is consistent with our findings from steady-state assays.

#### H3 acetylation after K14 (H3K14ac as a substrate)

To better understand the specificity of Gcn5, we utilized H3K14ac as a substrate under steady-state conditions. We hypothesized that while H3K14ac may be critical for transcription, secondary acetylation sites may also play a critical role in biology by improving the binding of bromodomains or competing with methylation (e.g. H3K9) which is critical for gene silencing [35]. This hypothesis is supported by the fact that deletions of gcn5 in vivo result in a loss of acetylation in residues acetylated after K14 [17], . These data suggest that while multiple enzymes (Gcn5, p300/CBP, etc) can acetylate K14 during transcription, Gcn5 may uniquely acetylate chromatin by altering other locations.

**Figure 7:**
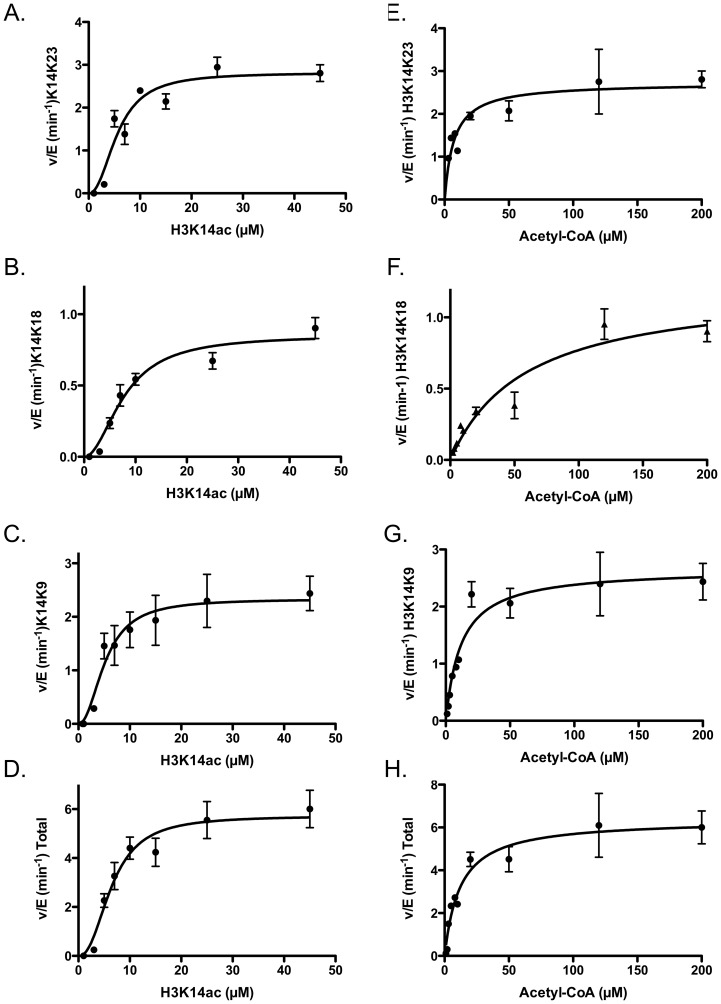
Determination of steady-state kinetic parameters of Gcn5-mediated acetylation of H3K14ac for individual and total lysine residues (i.e. K9, K18, and K23). The left panels (A)-(D) show the data when titrating H3K14ac; and the right panels (E)-(H) show the data when titrating acetyl-CoA. The apparent kinetic parameters are listed in Table 2. When fitting data from (A) to (D), the Hill coefficient (n_H_) is approximately equal to 2, suggesting H3K14ac could form a dimer to be further acetylated.

To test this hypothesis, we used H3K14ac as a starting substrate to determine the kinetic parameters for these second tier acetylation sites (Fig. 7 and Table 2 and 3). Using H3K14ac as a substrate, we observed five additional locations of acetylation: H3K9, K18, K23, K27, and K36. With five possible sites of acetylation, we had to monitor the sum of all acetylation sites to confirm that it was less than 10% of the total H3 location. Two sites, H3K9 and H3K23 had the highest level of acetylation, followed by H3K18, H3K27 and H3K36. From these data we calculated the steady-state parameters for H3K9, K18, and K23 (Fig. 7 and Table 2). We were unable to determine parameters for both K27 and K36, as we could not reach saturation for H3, but we did observe v/E rates as high as 2.0±0.5 min^−1^, which is faster than we would predict if the modification were simply due to nonenzymatic acetylation. Furthermore, in the time it took for total Gcn5 meditated acetylation to reach 10% we were unable to observe any acetylation on any residue in the absence of Gcn5.

The K_m(app)_or K_1/2(app.)_for H3K14ac for K9, K18, and K23 ranges from 5 to 7 µM and has a Hill coefficient of ∼2. It is possible that the Hill coefficient is reflecting the need for H3 to dimerism for efficient catalysis to occur. The largest difference is between k_cat(app)_ (K14ac substrate) where K9 and K23 are between 2–3 min^−1^ and K18 is approximately 1 min^−1^ (Table 3). The parameters for acetyl-CoA followed a similar trend but did not require the use of a Hill coefficient. The K_m(app)_ for acetyl-CoA for K9 and K23 were between 7–12 µM but H3K18 was >5-fold larger (59±8 µM), suggesting it would be the most sensitive to changes in acetyl-CoA levels followed by K9 and K23 (Fig. 7 and Table 2). Single turnover was not informative due to the fact that we could not reach the saturation, consistent with the higher observed K_1/2(app)_. Together these data are consistent with Gcn5 having a much lower efficiency of acetylating these positions relative to K14.

### Quantitating specificity and selectivity of multiple acetylation sites on a single protein

The specificity of histone acetylation is the ability of a KAT to acetylate a specific lysine. Selectivity is the ability of a KAT to acetylate one lysine relative to another. Specificity for a particular substrate is often defined as its catalytic efficiency or specificity constant (k_cat_/K_m_)[38]. Selectivity is expressed as the catalytic efficiency of one substrate to another by the ratio k_cat_/K_m_ of two substrates [39]–[41] or as the difference in catalytic proficiency which is the ratio of k_cat_/K_m_ to nonenzymatic acetylation rate, k_nE_ [42], [43]. Both k_cat_/K_m_ and k_nE_ are second order rate constants (µM^−1^ min^−1^) and thus both catalytic proficiency ((k_cat_/K_m_)/k_nE_) and the ratio of two specificity constants ((k_cat_/K_m_)_A_/(k_cat_/K_m_)_B_) have no units. For this reason we can calculate ΔΔG for these values. In this instance, both catalytic proficiency ((k_cat_/K_m_)/k_nE_) and ΔΔG relate the ability of Gcn5 to acetylate a specific residue relative to the fastest rate any residue can be acetylated nonenzymatically. Selectivity can also be easily obtained simply by taking the difference of ΔΔGs of two different residues, and this value equals the ΔΔG of selectivity calculated by –RTln((k_cat_/K_m_)_A_/(k_cat_/K_m_)_B_).

We calculated k_cat_/K_m_, (k_cat_/K_m_)/k_nE_, and ΔΔG for residues K9, K14, K18, and K23 for acetyl-CoA depend acetylation of H3 and H3K14ac by Gcn5. Using H3 as a substrate, we demonstrate a 2.4 million-fold ((2.43±0.49)×10^6^) increase in catalytic efficiency of K14 acetylation as compared to the fastest site of nonezymatic acetylation (K36) (Table 2). While a million-fold enhancement is consistent with other enzymes such as a carbonic anhydrase or chorismate mutase [43], [44], the rate of nonenzymatic acetylation of K36 is much faster than the nonenzymatic acetylation of K14 and therefore this rate is an underestimate of enhancement for this site. This does provide good insight into the level of specificity for K14 acetylation achieved by Gcn5. In order to measure the specificity and selectivity of sites other than K14ac we used H3K14ac as a substrate. While we observe acetylation of these sites with H3 as a substrate, they are too low too quantitate prior to 10% total acetylation, and single turnover suggests the K14ac is completely acetylated before any other residues. We measured catalytic proficiency for acetyl-CoA from 10^3^–10^6^ or −4.9 to −9.1 kcal/mol. Together these data suggest over a million-fold increase in specificity and up to a 1000-fold difference in selectivity between K14 and K18, the lowest measured catalytic proficiency. In addition, this selectivity is two orders of magnitude less than what is achieved by serine proteases [43] consistent with multiple sites being acetylated.

#### Specificity and selectivity in cooperative systems

We also measure the steady-state parameters for H3 and H3K14ac dependent acetylation by Gcn5. The acetylation of H3 was hyperbolic and displayed similar catalytic proficiency for K14 as acetyl-CoA (3.54±0.48×106). However, the acetylation of H3K14ac (substrate) was cooperative and as such does not follow the classical Michaelis-Menten equation (Table 3). In this case both the Km(app) or K1/2 and the Hill coefficient need to be considered to understand specificity. For this (kcat/Km) is replaced by (kcat/K1/2nH), where nH is the Hill coefficient [38]. While this analysis can be used to describe specificity and selectivity, it cannot be used to calculate a ΔΔG because both (kcat/K1/2nH)A/(kcat/K1/2nH)B and (kcat/K1/2nH)/knE have units of min-1µM-nHA+nHB or min-1µM-nH+1 respectively [38]. A Hill coefficient ∼2 makes a ∼6-11-fold difference in catalytic proficiency and efficiency and up to 100-fold in (kcat/Km)K14/(kcat/Km)X but makes little to no difference in selectivity between residues K9, K18, and K23 due the fact that these residues all display similar Hill coefficients.

## Discussion

The goal of this work was to lay the experimental and theoretical foundation to quantitate the specificity and selectivity of enzymes such as Gcn5, which can modify multiple lysines on a single protein substrate. The ability of an enzyme to modify multiple locations on a single protein suggests the potential of controlling multiple cell signals. This concept is similar to what in electronics would be called a multiplexer or a device that can send multiple signals down a single wire. The difficulty in studying a biological multiplexer is isolating a specific signal or modification. To solve this problem, we have developed an assay capable of monitoring all of the positions that can be acetylated by Gcn5, and in doing so we have demonstrated that label-free quantitative high throughput MS is a valuable quantitative method for studying enzyme kinetics. This assay has the advantage of not requiring isotopic reagents, can work with histones in any form from free in solution to nucleosomes, and is not hindered by multiple acetylation sites on a single histone.

Our first step to understanding the specificity of Gcn5 was simply to look at a full time course under steady-state conditions where histone H3 and acetyl-CoA were saturating. Under these conditions, we observed multiple positions of acetylation, but the highest efficiency was obviously K14, which is consistent with published results [17]. In addition, under conditions where only 10% of H3 is acetylated, K14 is the only measurable site of acetylation. We did not observe any acetylation of K9 in the absence of K14ac, even when K14 is more than 10% acetylated. We hypothesize that this is due to a much higher specificity for K14 and/or a dependence on K14ac. Similarly, K18 acetylation was followed by K23 acetylation, although 1–2% K18 acetylation was detected without K23 acetylation. Together this evidence strongly suggests that Gcn5 does not provide a random acetylation pattern *in vitro*. With H3 as a substrate, Gcn5 preferentially acetylates K14> K9 ≈ K23> K18> K27≈ K36. These data also demonstrate label-free quantitative MS can be easily applied to steady-state and single turnover approaches.

With multiple sites of acetylation, we needed a reference point for comparison. The most logical reference point is to compare catalytic proficiency or (k_cat_/K_m_)/k_nE_. While we were able to measure the second order rate constant for K36, other sites were difficult due to their low level of acetylation. Given that our goal is to understand the specificity of acetylation, we chose to use the rate of K36 nonenzymatic acetylation of H3 to represent the fastest possible rate a residue could be acetylated nonenzymatically. In this way, the catalytic proficiency reflects the ability of the enzyme to acetylate a specific residue relative to nonenzymatic acetylation on H3. This also allows for easy comparison because the selectivity is the difference in the ??G (catalytic proficiency) between residues and is the same as the ??G calculated by using ratio of k_cat_/K_m_ for two different residues.

We found a surprising degree of specificity to the nonenzymatic acetylation, with K36 being more acetylated than other positions. Although it is difficult to know if the nonenzymatic modification actually has a significant biological role, it has been found that nonenzymatic methylation plays a role in protein methylation of older human crystalline lens [45]. Given the rate of acetylation for H3K36, the reported half-life of histones in heterochromatin ranging from ∼100 to ∼600 days [46], [47] and H3 concentrations of ∼0.1 mM, nonenzymatic acetylation could be a possibility *in vivo*. While there is no definitive proof that nonenzymatic acetylation occurs *in vivo,* it has been suggested in mitochondria where high levels of acetylation are observed in the absence of any known KAT [48], [49]. In addition to H3 nonenzymatic acetylation has also been demonstrated on other proteins *in vitro* such as HIV-1 Tat, a protein that interacts with p300 [50].

Under the given conditions, we observed that K14 is the primary acetylation site for Gcn5. However, sites acetylated after K14 are difficult to characterize because when the majority of the substrate is H3K14ac, there is still a minor portion of unacetylated H3 and it would be difficult to calculate the time delay between the production of H3K14ac and the acetylation of the other sites. To solve this problem, we needed to start with H3K14ac, which can be considered a natural substrate given the higher efficiency of K14ac production and Gcn5's multiple roles *in vivo* [35], [51]. Therefore, starting with H3K14ac as a substrate will still provide a biologically relevant mechanism. These data suggest that under saturating conditions of acetyl-CoA and H3K14ac, K9 and K23 can be acetylated with approximately equal efficiency. But the acetylation of any secondary site is more dependent on acetyl-CoA concentration as reflected by the lower catalytic proficiency and efficiency. Consistent with this idea is the fact that the acetyl-CoA induced activation of SAGA (a complex which contains Gcn5) *in vivo* results in an increase of acetylation at K9, K14, K18, K23, and K27 of H3, but that H3K14 is the least and K18 is the most dependent on acetyl-CoA concentration [52]. We observed a change in specificity between saturating H3 and acetyl-CoA for H3K18ac possibly due to the difficulties in measuring extremely low rates of turnover as compared to K9 and K23. While we can observe acetylation on residues K27 and K36, we do not reach saturation on these positions under testable concentrations of H3. We hypothesize that the observed change in catalytic efficiency might reflect a way in which the cell can act to block K9 methylation under conditions of high metabolic activity or allow methylation in case of starvation.

The K_m_ of H3K14ac is less than that reported by Tanner and colleagues using calf thymus core histones [53] and this is likely due to different experimental conditions. However, a model of multiple competitive acetylation sites on a single substrate might explain this difference (Fig. 1). This model predicts that it would require a greater than 100-fold difference between either k_cat_ or K_m_ for H3K14 and the other locations before total acetylation would reflect acetylation for H3K14ac. Under our conditions we observe only a difference of 2–15-fold in k_cat_ and a 10-15-fold difference in K_m_, suggesting that a portion of the measured total acetylation is from H3K9, H3K18, and H3K23. It is important to note that we observe the same ternary complex formation mechanism for H3K14 acetylation (data not shown) consistent with that proposed by Tanner and colleagues [53]. The critical difference between our studies and others is that we are focused on understanding specificity from a residue specific level. In the case of multiple competitive acetylation sites on a single substrate (eq. 3, 4, and 5), each location of acetylation can influence the individual observed k_cat_ and K_m_ values of other locations of acetylation but not (k_cat_/K_m_)_(app)_ (eq. 6). Therefore, as long as the acetylation of a specific site is being measured and the initial rate is measured prior to 10% of the total substrate being acetylated at any site, the observed parameters of (k_cat_/K_m_)_(app)_ are unaffected by other sites of acetylation.

In this paper we have demonstrated the use of a label-free high throughput assay capable of quantitatively monitoring all of the acetylated positions on histone H3. While this assay is compatible with histones obtained from any source, the throughput makes it an ideal assays for studying enzymes which can modify multiple positions on the same histone. Using this assay we have characterized the selectivity and specificity of both the nonenzymatic and Gcn5 (enzymatic) mediated acetylation. In doing so we have shown that catalytic efficiently for both nonenzymatic and enzymatic acetylation is residue dependent. Furthermore, while it is known that altering the intracellular acetyl-CoA levels reduces histone acetylation in cells [18],our data suggest that this change will have a larger effect on secondary sites of acetylation with K_m(app)_ increasing as much as 80-fold. This approach provides a means to begin understanding the mechanism of acetylation and how a few key enzymes modify diverse sites. It is certainly interesting to speculate that the critical differences between KATs are due to their secondary acetylation sites in addition to the primary site, which must be acetylated to initiate transcription. This would imply that the KATs are capable of leaving signals as to which complex activates transcription and/or which gene should be blocked from silencing.
