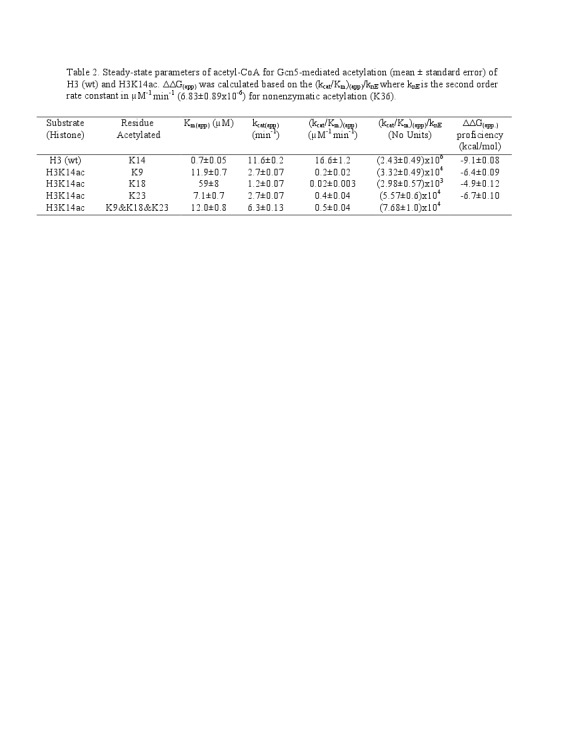# Correction: Quantitating the Specificity and Selectivity of Gcn5-Mediated Acetylation of Histone H3

**DOI:** 10.1371/annotation/b2bf9c2e-90a9-4228-9b38-2f1bc977a437

**Published:** 2013-10-23

**Authors:** Yin-Ming Kuo, Andrew J. Andrews

Table 3 was incorrectly uploaded as Table 2 and Table 3. The correct Table 2 can be found here: 

**Figure:**